# Epigenome-wide mediation analysis of the relationship between psychosocial stress and cardiometabolic risk factors in the Health and Retirement Study (HRS)

**DOI:** 10.1186/s13148-024-01799-4

**Published:** 2024-12-18

**Authors:** Lauren A. Opsasnick, Wei Zhao, Scott M. Ratliff, Jiacong Du, Jessica D. Faul, Lauren L. Schmitz, Xiang Zhou, Belinda L. Needham, Jennifer A. Smith

**Affiliations:** 1 Department of Epidemiology, School of Public Health, University of Michigan, 1415 Washington Heights, Ann Arbor, MI 48109 USA; 2 Survey Research Center, Institute for Social Research, University of Michigan, Ann Arbor, MI USA; 3 Department of Biostatistics, School of Public Health, University of Michigan, Ann Arbor, MI USA; 4 Robert M. La Follette School of Public Affairs, University of Wisconsin-Madison, Madison, WI USA

**Keywords:** Social epigenomics, Psychosocial stress, Cardiometabolic risk factors, Cardiovascular disease, Epigenome-wide mediation analysis, DNA methylation

## Abstract

**Background:**

Exposure to psychosocial stress is linked to a variety of negative health outcomes, including cardiovascular disease and its cardiometabolic risk factors. DNA methylation has been associated with both psychosocial stress and cardiometabolic disease; however, little is known about the mediating role of DNA methylation on the association between stress and cardiometabolic risk. Thus, using the high-dimensional mediation testing method, we conducted an epigenome-wide mediation analysis of the relationship between psychosocial stress and ten cardiometabolic risk factors in a multi-racial/ethnic population of older adults (*n* = 2668) from the Health and Retirement Study (mean age = 70.4 years).

**Results:**

A total of 50, 46, 7, and 12 CpG sites across the epigenome mediated the total effects of stress on body mass index, waist circumference, high-density lipoprotein cholesterol, and C-reactive protein, respectively. When reducing the dimensionality of the CpG mediators to their top 10 uncorrelated principal components (PC), the cumulative effect of the PCs explained between 35.8 and 46.3% of these associations.

**Conclusions:**

A subset of the mediating CpG sites were associated with the expression of genes enriched in pathways related to cytokine binding and receptor activity, as well as neuron development. Findings from this study help to elucidate the underlying mechanisms through which DNA methylation partially mediates the relationship between psychosocial stress and cardiometabolic risk factors.

**Supplementary Information:**

The online version contains supplementary material available at 10.1186/s13148-024-01799-4.

## Background

Cardiovascular disease (CVD), which is a class of disorders affecting the heart and blood vessels [1, 2], is the leading cause of death in the USA, accounting for nearly 930,000 deaths in 2020 alone [3]. Due to the high rates of CVD and subsequent CVD-related mortality in the USA and globally, substantial research has been conducted to understand risk factors associated with this disease. Several studies found that individuals at risk of CVD may have a cluster of cardiometabolic risk factors, which include hypertension, dyslipidemia, dysglycemia, and obesity [4]. Aside from these traditional risk factors, emerging evidence has shown that inflammatory profiles are independent cardiometabolic risk factors for CVD [5, 6], including increased levels of C-reactive protein (CRP), a sensitive and non-specific marker of inflammation produced in the liver [7, 8].

Psychosocial stress, defined as the perception of threat that may result in discomfort and emotional strain [9], is a well-established determinant of cardiometabolic risk [10, 11]. Several studies have identified associations between psychosocial stress and traditional cardiometabolic risk factors including hypertension [12], dyslipidemia [13], dysglycemia [14], and obesity [15], as well as CRP [16]. Additionally, prior research has linked psychosocial stress to epigenetic mechanisms, including DNA methylation, a biochemical modification to DNA and its related proteins that regulates gene expression without altering the underlying genetic sequence [17]. Both candidate gene studies and epigenome-wide association studies (EWAS) have examined the associations between psychosocial stress and DNA methylation [18–23]. Results from these studies showed relationships between psychosocial stress and CpG sites that mapped to genes associated with inflammation, hypertension, and coronary heart disease.

DNA methylation is also associated with the development of cardiometabolic risk factors. Large-scale EWAS have identified associations between individual CpGs and blood pressure [24], body mass index (BMI) [25], blood lipid levels [26], and CRP [27], and some of the identified CpG sites have a potential causal role in regulating genes that may influence the development of these cardiometabolic risk factors [28]. Thus, DNA methylation may act as both an upstream regulator and a downstream marker of cardiometabolic processes. Limited research suggests that DNA methylation may mediate the relationship between individual psychosocial stressors, including neighborhood disadvantage, prenatal adversity, and childhood trauma, and cardiometabolic risk [29–31]. However, to our knowledge, no study has examined the mediating effect of DNA methylation across the genome on the association between cumulative psychosocial stress across the life course and cardiometabolic risk factors.

In this study, we conducted a high-dimensional mediation analysis to identify whether DNA methylation mediates the relationship between cumulative psychosocial stress and ten cardiometabolic risk factors. Further, we tested for enrichment of functional elements and biological pathways of the mediating CpG sites to investigate the functional role of identified genes and better understand the mechanisms through which stress may influence cardiometabolic processes in older adults.

## Methods

### Study sample

Participant data are from the Health and Retirement Study (HRS), a longitudinal cohort comprised of approximately 20,000 participants in each wave that is nationally representative of Americans over age 50. Detailed information regarding the HRS protocol and study design can be found elsewhere [32, 33]. In brief, HRS, which originated in 1992, consists of biennial surveys that collect information regarding income, employment, physical and mental health, and cognitive functioning. Beginning in 2006, a random one-half of the HRS sample was selected to complete an enhanced face-to-face interview, which captured participants’ physical and biological measures, including blood pressure, height, weight, and waist circumference [34]. Participants who complete the enhanced face-to-face interview are also given the Psychosocial and Lifestyle Questionnaire, a self-administered psychosocial survey capturing information on participants’ well-being, lifestyle, and social support [35]. In 2008, an enhanced face-to-face interview was administered to the remaining half of the sample. Data from the enhanced interview are available longitudinally every four years for all participants.

**Fig. 1:**
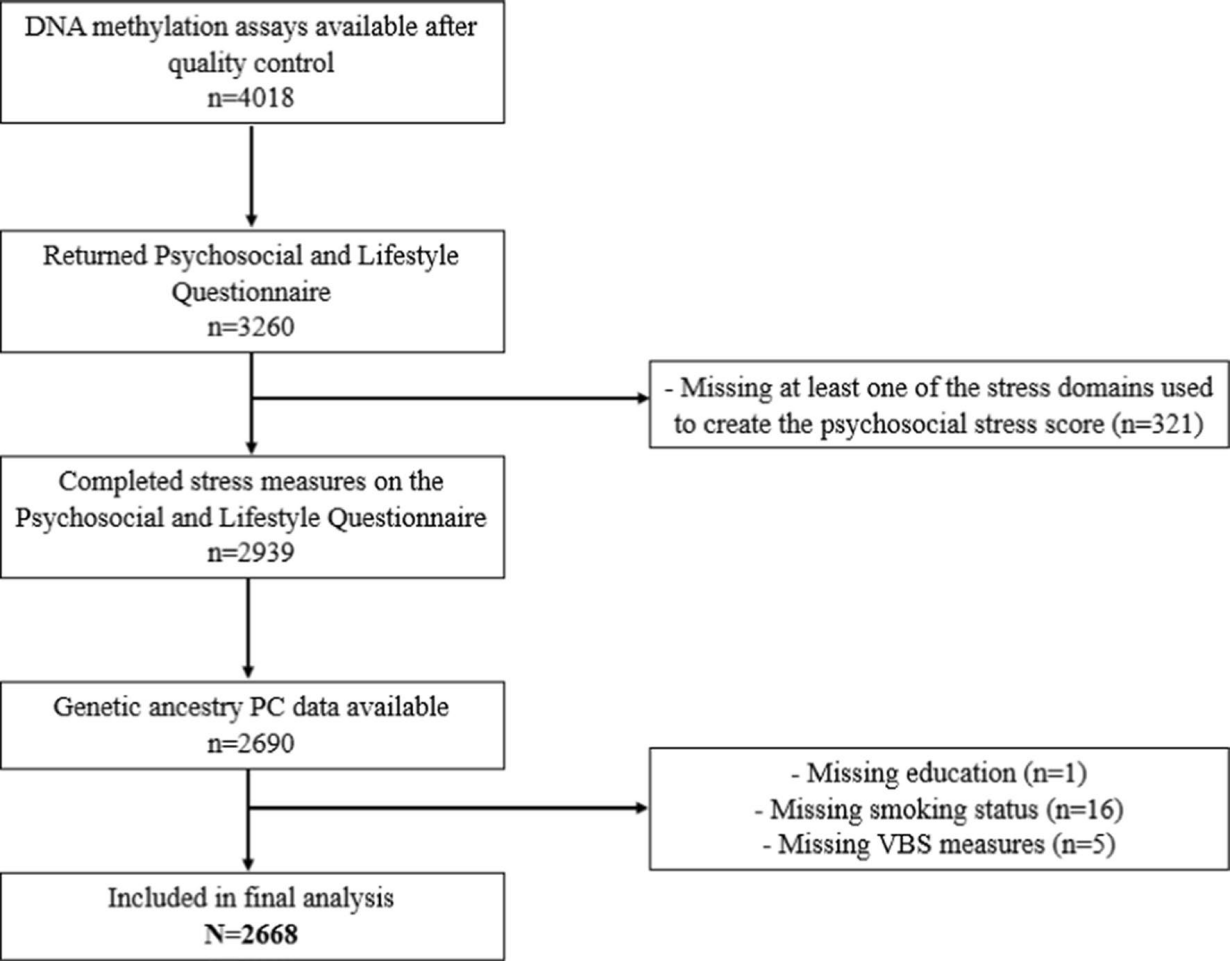
Flow diagram detailing participant inclusion criteria for epigenome-wide mediation analyses

Additionally, all panel respondents who completed an HRS interview in 2016 were invited to participate in an ancillary study, the Venous Blood Study (VBS, *n* = 9934), where blood-based biomarkers were assayed [36]. DNA methylation was measured in a sample of participants who completed the VBS blood draw (*n* = 4018). Of the 4018 participants who had DNA methylation data, 3260 completed and returned the Psychosocial and Lifestyle Questionnaire. Subsequently, 343 participants were removed due to missing data on relevant psychosocial stress, sociodemographic, or blood biomarker variables, leaving 2668 participants in the final analytic sample. A flow diagram detailing participant inclusion criteria is provided in Fig. 1.

To understand how using complete cases data impacts the representativeness of the final sample, we compared participant characteristics between those who were included (*n* = 2668) and excluded (*n* = 1350) in the analysis from the total DNA methylation sample. Chi-squared tests and Student’s t-tests were used, as appropriate. Further, to quantify the size of the difference between the two groups, Cramer’s V and Cohen’s d effect sizes were calculated for categorical and continuous variables, respectively.

### DNA methylation

DNA methylation data from HRS were assessed from whole blood samples using the Illumina Infinium HumanMethylationEPIC V 1.0 chip. Samples were randomized across plates by key demographic variables, including age, cohort, sex, education, and race/ethnicity with 39 pairs of blinded duplicates [37]. The *minfi* R package was used for data preprocessing and quality control, resulting in the removal of 3.4% of methylation probes due to their detection p value falling below the threshold of 0.01 (*n* = 29,431 out of 866,091) [38]. Sex mismatched samples and any controls (cell lines, blinded duplicates) were removed from the analysis, as were probes with missing data for > 5% of the sample. Additionally, we removed cross-reactive probes that target repetitive sequences, potentially mapping to multiple places in the genome [39]. Missing DNA methylation data was imputed using the mean of each CpG [40]. Finally, to ensure that strong outliers were not impacting the results, we winsorized methylation values greater than 3 times the interquartile range (IQR) of the 25th and the 75th percentiles [41, 42]. In total, 789,656 CpGs were available for analysis after quality control. DNA methylation was quantified using beta values, which approximate the proportion of methylation, making them biologically interpretable. To account for cell-type heterogeneity, we pre-adjusted DNA methylation by regressing each methylation beta value on white blood cell proportions estimated by the Houseman method [43]. Additionally, methylation sample plate and position (sample plate row and column) were added as random effects to account for potential batch effects.

### Psychosocial stress

Data from the Psychosocial and Lifestyle Questionnaires were used to construct a cumulative psychosocial stress score [35]. Because this survey was administered to only half the HRS participants at each wave, we utilized data from both the 2010 and 2012 psychosocial surveys to ensure all participants had the opportunity to respond. Furthermore, we measured psychosocial stress at these specific timepoints as they were the only set of two consecutive timepoints that captured all of the individual stress measures needed to create the cumulative stress score.

Based on previous HRS studies, we examined six domains of psychosocial stress: (1) acute life events, (2) financial stress, (3) neighborhood stress, (4) relationship stress, (5) lifetime discrimination, and (6) childhood adversity [23, 44, 45]. Because the focus of this study was to understand how lifetime stress exposure affects DNA methylation and subsequent cardiometabolic risk, we evaluated both acute and chronic stressors spanning the life course. Each domain included one or more individual stress measures, detailed below and in Additional file 1: Table S1. For an individual measure to be considered complete, participants had to have answered at least 80% of the questions within the measure, unless otherwise noted in the measurement scoring instructions [46]. Only participants who had completed all stress measures were included in the analysis. Each stress measure was transformed into a z-score. For domains comprised of multiple stress measures, z-scores from individual measures were summed together and subsequently standardized, allowing for cross-domain comparisons [47, 48].

The six domains of stress are as follows: (1) *Acute life events,* which included two measures: (i) acute lifetime traumas (seven items) and (ii) acute stressful life events in the past 5 years (six items); (2) *Financial stress*, which included two measures: (i) financial strain (two items) and (ii) lack of financial autonomy (two items); *Neighborhood stress*, included one measure (four items), related to neighborhood safety, vandalism, and cleanliness; *Relationship stress*, which included four measures: (i) spouses (four items), (ii) children (four items), (iii) other family members (four items), and (iv) friends (four items); (5) *Lifetime discrimination*, which included two measures: (i) perceived everyday discrimination (five items) and (ii) major discriminatory events (six items); and (6) *Childhood adversity*, which included one measure related to lifetime traumas in adolescence (four items).

Finally, we calculated a cumulative psychosocial stress score by summing the standardized scores from each of the six domains and re-standardizing the summed scores [44]. This cumulative score represents the total stress exposure across all stress domains, and it is the primary exposure of interest in this study.

### Cardiometabolic risk factors

We examined ten cardiometabolic risk factors, including body mass index (BMI), waist circumference (WC), systolic blood pressure (SBP), diastolic blood pressure (DBP), glucose, total cholesterol (TC), low-density lipoprotein cholesterol (LDL-C), high-density lipoprotein cholesterol (HDL-C), triglycerides (TG), and high-sensitivity C-reactive protein (CRP). Measurement methods have been described elsewhere [34, 36]. Glucose, TC, LDL-C, HDL-C, TG, and CRP measurements were collected from the VBS blood draw (2016). Fasting was recommended, but not required, and participant’s fasting status was recorded by the phlebotomist. We log-transformed glucose and CRP measures due to the skewed nature of the data. BMI, WC, SBP, and DBP were measured during the participant’s enhanced face-to-face interview (2016 or 2018). BMI was calculated from the participant’s weight in kilograms divided by the square of their height in meters. For participants who did not have their height and weight measured during the enhanced interview, we used their self-reported BMI.

For each cardiometabolic risk factor, we removed extreme outliers, defined as values greater than 3 times the IQR of the 25th and the 75th percentiles. Additionally, we adjusted select cardiometabolic risk factors for participant medication use, as appropriate, based on previously reported studies. For participants who self-reported taking hypertension medication, we added 15 Hg and 10 Hg to their SBP and DBP measures, respectively [24, 49]. For participants who reported using statin medication, we divided their LDL-C levels by 0.7 and their TC levels by 0.8 [50, 51]. HDL-C and TG were not adjusted for lipid-lowering medication use [51, 52]. Finally, when assessing the total effect of psychosocial stress on glucose, we removed any participants who self-reported use of diabetes medication [53, 54].

### Covariates

Sociodemographic characteristics were collected at the 2010 or 2012 interview, concurrent with the timing of the participant’s psychosocial stress assessment. These variables included: age, sex (male or female), educational attainment (no degree, high school degree, college degree, or higher), and smoking status (never, former, current). Additionally, the top 10 principal components (PCs) of genetic ancestry, which were estimated using genome-wide genotype data [55], were used as model covariates to account for population stratification and admixture.

### Statistical analysis

#### Associations between psychosocial stress and cardiometabolic risk factors

We first examined whether cumulative psychosocial stress was associated with the ten cardiometabolic risk factors (total effects), using linear regression models adjusted for age, sex, educational attainment, year of psychosocial stress measurement, and genetic ancestry PCs; fasting status was also included in models for glucose, HDL-C, LDL-C, and TG (Model 1). Furthermore, since smoking is a known risk factor for cardiometabolic disease and may be on the causal pathway between psychosocial stress and cardiometabolic risk factors, we were interested in the associations between stress and cardiometabolic risk both before and after controlling for any mediating effects of smoking. Thus, we further adjusted the total effect models for smoking (Model 2). Associations between psychosocial stress and cardiometabolic risk factors with *P* < 0.05 in Model 1 were selected for mediation analysis.

Additionally, because fasting status is known to greatly affect the measurement of certain blood-based cardiometabolic risk factors, we conducted a sensitivity analysis to examine the associations between cumulative psychosocial stress and glucose, HDL-C, LDL-C, and TG, restricting the sample to participants who fasted for their VBS blood draw.

### Mediation analysis

For significant associations identified between psychosocial stress and cardiometabolic risk factors, we conducted an epigenome-wide mediation analysis to identify CpG sites that may mediate the relationship between stress and the relevant risk factors. The mediation effect was defined as the product of the exposure-mediator effect (*α*) and mediator-outcome effect (*β*$$)$$, adjusting for the exposure [56]. We used regression models to estimate: (1) the association of psychosocial stress with each DNA methylation site (Eq. 1) and (2) the association of each DNA methylation site with each cardiometabolic risk factor, adjusting for psychosocial stress (Eq. 2).

1 $$M_{j} = \alpha_{0,j} + \alpha_{j} X + \alpha_{C,j}^{T} C + \varepsilon_{M,j } ,$$

2 $$Y = \beta_{0,j} + \beta_{X,j} X + \beta_{j} M_{j} + \beta_{C,j}^{T} C + \varepsilon_{Y, j} .$$

In these equations, which represent the exposure-mediator and mediator-outcome relationships for a single individual, *M*_*j*_ represents DNA methylation (beta value) for *j* = 1,2…*J* mediators, *X* represents cumulative psychosocial stress, *C* is the set of potential confounders (age, sex, educational attainment, 10 genetic ancestry PCs), *Y* represents a single cardiometabolic risk factor, and ${\varepsilon }_{M,j}$ and ${\varepsilon }_{Y, j}$ are residual errors, following an independent and normal distribution.

We evaluated the epigenetic mediation effect by testing: ${H}_{0,j}: {\alpha }_{j}* {\beta }_{j}=0$ vs. ${H}_{1,j}: {\alpha }_{j}* {\beta }_{j} \ne 0$ for j = 1,2,…J mediators using the high-dimensional mediation testing (HDMT) method. This method uses a corrected reference distribution for the MaxP statistic, $\text{max}({p}_{{\alpha }_{j}},{p}_{{\beta }_{j}})$, with ${p}_{{\alpha }_{j}}$ and ${p}_{{\beta }_{j}}$ representing the p values for testing ${\alpha }_{j}=0$ and ${\beta }_{j}=0$ in Eqs. 1 and 2, respectively [57]. HDMT accounts for the compositional nature of the null hypothesis through a mixture reference distribution. In low-power settings (i.e., over half a million CpG sites being investigated, each likely with a small mediation effect), HDMT is advantageous because it increases power by providing finite sample-size adjustment for p values. Additionally, this method controls for false positive rates while also yielding high true positive rates better than other high-dimensional mediation methods that do not use a mixture reference distribution, including Sobel’s test and MaxP test [58]. Epigenome-wide mediation analysis was conducted using the *medScan* package in R. We corrected for multiple testing using the false discovery rate (FDR), where a CpG was considered a mediating site if FDR *q* < 0.05.

Due to the possible correlation between CpGs within close proximity to one another, we were unable to quantify the overall mediation effects of each set of CpG mediators using the HDMT method. Thus, we performed a PC analysis on the identified CpG mediators for each cardiometabolic risk factor [29]. PC analysis reduces the dimensionality of the CpG mediators and generates methylation PCs that are uncorrelated. Therefore, the cumulative effect of the top PCs represents the lower bound of the mediation effect across all CpG mediators. After calculating the top 10 PCs for each set of CpG mediators, we used the *mediate* package in R to test and estimate the mediation effect of each methylation PC individually [59]. Using nonparametric bootstrapping with 10,000 iterations, we obtained the average mediation effect estimate, as well as the proportion of the total effect explained by each methylation PC.

Additionally, for each set of CpG mediators identified in HDMT, we conducted a penalized regression-based high-dimensional mediation analysis using the *hima2()* function in R [60], which allowed us to determine the CpG mediators that remain significant after adjusting for all other mediating CpGs. For each cardiometabolic risk factor, we fit a multivariable mediation model, assigning all CpGs identified in HDMT as the mediators. A de-biased LASSO regression was applied to estimate the regression parameters of each CpG site on a given cardiometabolic risk factor, controlling for the other CpGs in the model [61]. To determine the significance of the mediation effect, we performed a joint significance test with a mixture of null distributions from the exposure-mediator and mediator-outcome models to accurately control for the false discovery rate. A CpG site was considered significant when FDR *q* < 0.05.

Finally, to better understand the epigenetic mediation of the association between psychosocial stress and cardiometabolic risk factors independent of smoking, we conducted sensitivity analyses by further adjusting all HDMT, PC, and de-biased LASSO-based mediation models for smoking status.

### Functional characterization of CpG mediators

We performed genomic feature enrichment analysis to examine whether the locations of each set of mediating CpGs were enriched for genomic features, including gene promoters, enhancers, DNase I hypersensitivity sites (DHS), CpG islands (CGI), and CpG island flanking shores or shelves. CpG sites were considered to be in the promoter region if they were located less than 1.5 kb upstream of a transcriptional start site. CpGs were designated as CGI flanking shores or shelves if they were within 2 kb or between 2 and 4 kb of a CGI, respectively. We used annotation files from both Illumina and the UCSC genome browser to identify the proximal genes and genomic features associated with each CpG [62, 63].

Additionally, to assess whether the identified CpGs were associated with gene expression, we used empirical data from Framingham Heart Study (FHS) [64]. Keshawarz et al. (2023) performed expression quantitative trait methylation (eQTM) analysis to detect CpG sites whose methylation levels were associated with gene expression. In total, they identified 70,047 cis-eQTMs (CpG-transcript pairs located < 1 Mb apart) and 246,667 trans-eQTMs (CpG-transcript pairs located > 1 Mb apart) in whole blood samples at significance levels of P ≤ 1E−07 and P ≤ 1E−14, respectively. To test whether each set of mediating CpGs was more likely to be enriched as eQTMs, we compared the number of significant CpG sites that mapped to gene transcripts in the eQTMs versus the number of non-significant CpGs that mapped to transcripts. Enrichment analyses were performed using a two-sided Fisher’s exact test.

To better understand the underlying biological pathways of the CpG mediators, we conducted Gene Ontology (GO) and Kyoto Encyclopedia of Genes and Genomes (KEGG) pathway analyses for each cardiometabolic risk factor. We first performed a gene-set analysis using the gometh() function from the R package *missMethyl* [65]. This method accounts for potential sources of bias, including the uneven distribution of CpGs across the genome, as well as CpGs that are annotated to multiple genes. However, one constraint of this method is that it uses annotation files to map CpG sites to their proximal genes based on chromosomal position. Because CpGs do not always act on the nearest gene, but instead may affect the expression of distal genes, we performed a second enrichment analysis using enrichGO() and enrichKEGG() functions in the *clusterProfiler* R package [29, 66]. This method allows us to pre-define the gene list based on existing CpG-gene expression pairs, providing a more robust examination of functional pathways. Using the FHS eQTM dataset, genes known to be associated with each set of CpG mediators were extracted and used as the signal gene list. The background gene list included all genes associated with CpG sites that appear in both the FHS study and our study. GO terms and KEGG pathways with FDR *q* < 0.01 were considered significant for all gene-set analyses.

## Results

Sample characteristics are displayed in Table 1. The mean age of participants was 70.4 years (SD: 9.5) and the majority were female (59%). Over a quarter of participants had a college degree or higher (26%). Additionally, 74% of participants were non-Hispanic white, 13% were non-Hispanic Black, 10% were Hispanic, and 3% were another race or ethnicity. Approximately 45% of the participants never smoked, while 43% formerly smoked and 12% currently smoked at the time of interview. Half the participants reported taking lipid-lowering medication, 57% reported taking antihypertensive medication, and 21% reported taking diabetes medication. Participants from the DNA methylation sample with complete data on stress, blood biomarkers, and covariates were older, had higher educational attainment, and had healthier smoking habits than those who were excluded; however, the effect sizes were relatively small, with a Cramer’s V and Cohen’s *d* < 0.30 for all covariates (Additional file 1: Table S2).

**Table 1:** Characteristics of the Health and Retirement Study analytic sample (*N* = 2668)

Characteristics		*N* (%) or mean (SD)
Age, years		70.4 (9.5)
Female		1575 (59.0)
*Race/ethnicity*		
Hispanic		269 (10.1)
Black		349 (13.1)
White		1981 (74.3)
Other		68 (2.5)
*Education*		
No degree		346 (13.0)
HS degree		1617 (60.6)
College degree or higher		705 (26.4)
*Smoking status*		
Never smoker		1207 (45.2)
Former smoker		1134 (42.5)
Current smoker		327 (12.3)
*Medication use*		
Lipid-lowering medication (*n* = 2665)		1335 (50.1)
Antihypertensive medication (*n* = 2352)		1354 (57.6)
Diabetes medication (*n* = 2654)		546 (20.6)
*Cardiometabolic risk factors*		
Body mass index (kg/m^2^, *n* = 2537)		30.1 (6.7)
Waist circumference (cm, *n* = 2346)		40.8 (6.2)
Glucose (mg/dL, *n* = 2100)		99.7 (21.3)
Systolic blood pressure (mm Hg, *n* = 2363)^a^		127.0 (17.9)
Diastolic blood pressure (mm Hg, *n* = 2363)^a^		75.7 (10.4)
Total cholesterol (mg/dL, *n* = 2664)^a^		186.8 (41.3)
High-density lipoprotein cholesterol (mg/dL)		57.7 (19.1)
Low-density lipoprotein cholesterol (mg/dL, *n* = 2611)^a^		100.6 (34.7)
Triglycerides (mg/dL, *n* = 2631)		138.7 (68.8)
C-reactive protein (mg/dL, *n* = 2666)		4.8 (10.1)

^a^Values reported are prior to adjustment for medication use

After adjusting for age, sex, education, year of psychosocial stress measurement, and top 10 genetic PCs, along with fasting status, when appropriate, an increase in cumulative psychosocial stress was associated with higher BMI, WC, DBP, and CRP, in addition to lower HDL-C (Model 1; Table 2). All of these associations remained significant after further adjusting for smoking (Model 2; Table 2).

**Table 2:** Total effect of cumulative psychosocial stress on cardiometabolic risk factors (*N* = 2668)

	Cumulative psychosocial stress			
	Model 1		Model 2	
Cardiometabolic risk factors	Beta	*P* value	Beta	*P* value
BMI	**0.80**	**1.16E−08**	**0.87**	**6.34E−10**
WC	**0.92**	**3.79E−12**	**0.95**	**1.28E−12**
SBP^a^	0.38	0.399	0.41	0.367
DBP^a^	**0.58**	**0.026**	**0.59**	**0.023**
Log (Glucose)^a,b^	0.006	0.181	0.006	0.191
TC^a,b^	**−** 1.74	0.058	**−** 1.71	0.064
LDL-C^a,b^	**−** 1.09	0.203	**−** 1.01	0.238
HDL-C^b^	**−** **1.48**	**7.67E−05**	**−** **1.44**	**1.25E−04**
TG^b^	2.70	0.056	2.56	0.071
Log (CRP)	**0.10**	**3.34E−06**	**0.10**	**3.68E−05**

*BMI* body mass index, *WC* waist circumference, *SBP* systolic blood pressure, *DBP* diastolic blood pressure, *TC* total cholesterol, *LDL-C* low-density lipoprotein cholesterol, *HDL-C* high-density lipoprotein cholesterol, *TG* triglycerides, *CRP* C-reactive protein

Model 1: Cardiometabolic Risk Factor ~ Cumulative Psychosocial Stress + Age + Sex + Education + Year of psychosocial stress measure + Top 10 genetic PCs

Model 2: Model 1 + Smoking

^a^Cardiometabolic risk factors were pre-adjusted for medication use (antihypertensive, lipid-lowering, and diabetes medication)

^b^Fasting status (yes/no) was included as an additional covariate in the models

Bold text indicates *P* < 0.05

When restricting the sample to only participants who fasted for the VBS blood draw, the associations between psychosocial stress and glucose, TC, HDL-C, LDL-C, and TG were substantively similar to results from the main total effect models (Additional file 1: Table S3).

For associations between stress and cardiometabolic risk factors with *p* < 0.05, we tested for epigenetic mediation, adjusting for the same covariates used in Model 1. We identified 50, 46, 7, and 12 CpGs that significantly mediated the associations between psychosocial stress and BMI, WC, HDL-C, and CRP, respectively (Additional file 2: Figures S1-S4). No CpGs were found to mediate the relationship between stress and DBP. A summary of the mediation results for each cardiometabolic risk factor can be found in Table 3.

**Table 3:** High-dimensional epigenetic mediation results of the association between psychosocial stress and cardiometabolic risk factors

Cardiometabolic Risk Factor	% variance explained by psychosocial stress^a^	# of mediating CpGs identified in HDMT^b^	% Mediated by individual CpGs in HDMT^b^	Cumulative % Mediated by methylation PCs^c^	CpGs identified in de-biased LASSO mediation analysis^d^	% Mediated by individual CpGs in LASSO^d^
BMI	16.5%	50	4.3–11.0%	46.3%	cg00420390, cg19748455, cg02370100, cg08366476, cg13770461, cg09885325, cg09971499, cg04881642, cg02508743	3.3–9.2%
WC	16.7%	46	4.2–10.6%	35.9%	cg19748455, cg11454468, cg03037271, cg02370100, cg04803208, cg00420390	3.7–8.4%
HDL-C	3.1%	7	9.4–14.5%	31.4%	cg04803208, cg25607249, cg08274633	6.8–13.0%
CRP	15.6%	12	6.5–11.4%	45.1%	cg02508743, cg03699074, cg00420390, cg15781610, cg26010590, cg11956636, cg24837149	4.0–9.3%

*BMI* body mass index, *WC* waist circumference, *HDL-C* high-density lipoprotein cholesterol, *CRP* C-reactive protein, *HDMT* high-dimensional mediation testing, *PC* principal component

All models were adjusted for Model 1 covariates: age, sex, education, year of psychosocial stress measure, top 10 genetic PCs, and fasting status (when appropriate)

^a^Calculated as the change in R-squared values from Model 1 with and without including the psychosocial stress variable divided by the R-squared value of Model 1 including the psychosocial stress variable

^b^Results from the epigenome-wide mediation analysis using the HDMT method. The percent mediated by each individual CpG was not controlled for the other mediating CpGs

^c^Results from the methylation PC analysis, which reduced the dimensionality of the CpGs identified in HDMT to top uncorrelated PCs. The cumulative percent mediated by all PCs approximates the lower bound of the overall mediation effect of the CpGs on the relationship between stress and each cardiometabolic risk factor

^d^Results from de-biased LASSO mediation analysis, which implemented a penalization-based approach to identify CpGs from HDMT that remained significant after adjusting for other mediating CpGs. The percent mediated by each individual CpG was controlled for the other mediating CpGs

Cumulative psychosocial stress explained 16.5% of the total variability in BMI after adjusting for age, sex, education, year of psychosocial stress measurement, and 10 genetic PCs. Through high-dimensional mediation testing, we identified 50 CpG sites that mediated between 4.3% and 11.0% of the total effect of stress on BMI. Complete summary statistics and gene annotations for these CpG mediators are provided in Additional file 1: Table S4. Additionally, Manhattan and QQ-plots from the epigenome-wide mediation analysis can be found in Additional file 2: Figure S1.

The first 10 epigenetic PCs accounted for 51.2% of the variability across the 50 mediating CpGs. After performing individual mediation analyses on each of the PCs, 5 of the 10 PCs (PC1, PC3, PC5, PC6, PC8) significantly mediated the association between stress and BMI, explaining between 2.0 and 25.4% of the total effect (Additional file 1: Table S5). Cumulatively, these 5 PCs mediated 46.3% of the association between stress and BMI. When performing the de-biased LASSO-based mediation model, 9 of the 50 CpG sites showed evidence of mediation after adjusting for the other CpG mediators (Additional file 1: Table S6). Individually, these 9 CpGs mediated between 3.3 and 9.2% of the total effect of psychosocial stress on BMI.

After adjusting for Model 1 covariates, cumulative psychosocial stress explained 16.7% of the total variability in WC. Between 4.2 and 10.6% of this total effect of stress on WC was mediated by 46 CpG sites (Additional file 1: Table S7). Corresponding Manhattan and QQ-plots can be found in Additional file 2: Figure S2.

The first 10 epigenetic PCs accounted for 55.7% of the variability across the 46 mediating CpGs. Five PCs (PC1, PC4, PC5, PC8, PC9) significantly mediated the association between psychosocial stress and WC, explaining between 1.8 and 17.5% of the total effect (Additional file 1: Table S8). Cumulatively, these 5 PCs mediated 35.9% of the relationship between psychosocial stress and WC. Results from the penalized-based high-dimensional mediation analysis showed that 6 of the 46 CpG sites identified through HDMT were mediators of the stress-WC association after adjusting for all other CpGs (Additional file 1: Table S9). Individually, these 6 CpGs mediated between 3.7 and 8.4% of the total effect of psychosocial stress on WC.

Cumulative psychosocial stress explained 3.1% of the total variability in HDL-C after adjusting for Model 1 covariates. Of this relatively small total effect, between 9.4% and 14.5% was mediated by 7 CpGs (Additional file 1: Table S10 and Additional file 2: Figure S3).

Due to the small number of mediating CpGs identified in the epigenome-wide mediation analysis, we extracted only the top 5 epigenetic PCs, which accounted for 87.7% of the variability across the 7 mediating CpGs. We found that PC1 and PC2 significantly mediated 22.9% and 8.5% of the association between stress and HDL-C, respectively (Additional file 1: Table S11). Cumulatively, 31.4% of the relationship between psychosocial stress and HDL-C was due to the epigenetic mediation of the 7 identified CpGs. In the penalized-based mediation analysis, 3 of the 7 CpGs showed evidence of mediation after adjusting for the other CpG mediators (Additional file 1: Table S12). Cg04803208, cg25607249, and cg08274633 mediated 13.0%, 8.4%, and 6.8% of the total effect of psychosocial stress on HDL-C, respectively.

After adjusting for Model 1 covariates, cumulative psychosocial stress explained 15.6% of the total variability in CRP. Of this total effect, between 6.5% and 11.4% was mediated by 12 CpG sites identified by HDMT (Additional file 1: Table S13 and Additional file 2: Figure S4).

The first 10 epigenetic PCs accounted for 92.3% of the variability across the 12 mediating CpGs. PC1, PC2, and PC3 significantly mediated the association between stress and CRP, explaining 27.5%, 14.0%, and 3.6% of the total effect, respectively (Additional file 1: Table S14). Cumulatively, 45.1% of the relationship between psychosocial stress and CRP could be attributed to epigenetic mediation by the 12 identified CpGs. Furthermore, results from the de-biased LASSO mediation analysis showed that 7 of the 12 CpG sites mediated between 4.0% and 9.3% of the relationship between psychosocial stress and CRP after adjusting for all other CpG mediators (Additional file 1: Table S15).

Because we were interested in the epigenetic mediation of the association between stress and cardiometabolic risk factors independent of smoking, we further adjusted all mediation models for smoking status. Compared to results from the primary epigenome-wide mediation models, the mediation effects of CpGs across all cardiometabolic outcomes were slightly attenuated after accounting for smoking; however, all CpGs remained nominally significant (p < 5.6E-05). Additionally, 25, 9, 3, and 2 CpGs found to mediate the relationships between psychosocial stress and BMI, WC, HDL-C, and CRP, respectively, remained FDR significant after adjusting for smoking (FDR *q* < 0.05). Further adjusting the methylation PC and penalized-based mediation models for smoking yielded substantively similar results across all cardiometabolic outcomes (Model 2; Additional file 1: Tables S4-15).

**Fig. 2:**
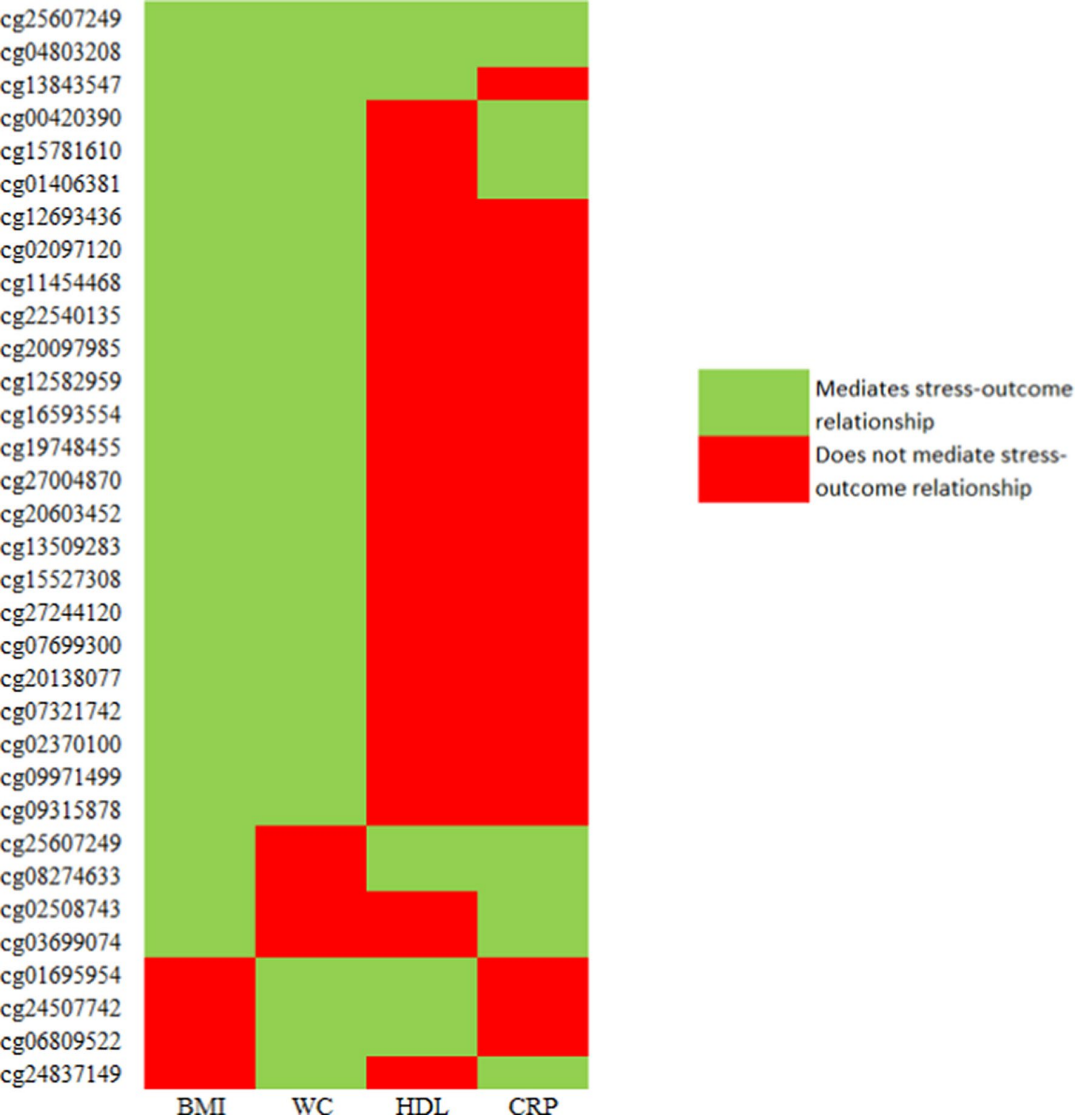
CpGs identified in HDMT that mediate the relationship between stress and multiple cardiometabolic risk factors. Abbreviations: Body mass index (BMI); Waist circumference (WC); High-density lipoprotein cholesterol (HDL-C); C-reactive protein (CRP); High-dimensional mediation testing (HDMT)

In total, we identified 75 distinct CpGs that mediated the relationship between stress and at least one cardiometabolic risk factor (Additional file 1: Table S16). Overall, 22, 17, and 4 of these CpGs uniquely mediated the relationships between stress and BMI, WC, and CRP, respectively. No CpGs exclusively mediated the association between stress and HDL-C. The remaining 32 CpGs were identified as mediators in at least two epigenome-wide mediation analyses (Fig. 2). Two CpGs in particular, cg04803208 and cg25607249, were found to mediate the relationship between stress and all four cardiometabolic risk factors.

Based on the Illumina annotation mapping, compared to non-mediating CpG sites, CpGs that mediated the relationships between psychosocial stress and BMI, WC, and CRP were more likely to reside in enhancer regions, suggesting these CpGs may be involved in the regulation of nearby gene expression (Table 4; all *p* < 0.001). We observed no significant enrichment of these CpGs in promoter regions, DHS, CGIs, or CGI flanking shores or shelves, nor did we detect enrichment of any genomic features for the CpGs that mediated the association between stress and HDL-C.

**Table 4:** Genomic feature enrichment of mediating CpG sites identified in epigenome-wide mediation analysis (model 1)

Genomic feature	BMI		WC		HDL-C		CRP	
	Odds ratio	*P* value	Odds ratio	*P* value	Odds ratio	*P* value	Odds ratio	*P* value
DHS	1.11	0.78	1.92	0.051	5.43	0.094	2.04	0.38
Enhancer	**5.59**	**0.0002**	**6.17**	**1.19E-04**	3.66	0.26	**9.77**	**0.006**
Promoter	0.82	0.74	0.49	0.12	2.46	0.21	0.66	0.74
Shore/shelf	0.92	0.99	1.03	0.87	2.20	0.38	2.09	0.20
CpG Island	0.46	0.11	0.40	0.091	0.52	0.98	0.32	0.33
eQTM	**9.67**	**5.03E-10**	**7.25**	**8.85E-07**	**8.22**	**0.039**	**14.67**	**1.30E-04**

*DHS* DNAse hypersensitivity site, *eQTM* expression quantitative trait methylation site

Bold text indicates associations present at *P* < 0.05

Overall, 36,662 of the 789,656 (4.6%) CpGs considered in the epigenome-wide mediation analysis mapped to at least one gene transcript using eQTM data from Keshawarz et al. (2023). In our study, 16 (36.0%), 12 (26.1%), 2 (28.6%), and 5 (41.7%) of the identified CpG mediators for BMI, WC, HDL-C, and CRP, respectively, mapped to gene transcripts in the eQTMs. Compared to the non-mediating CpG sites, CpG mediators of all four cardiometabolic risk factors were enriched for eQTMs, suggesting the mediating CpGs identified by HDMT were more likely to influence gene expression (Table 4; *p* < 0.05).

CpGs found to mediate the relationships between stress and BMI, WC, HDL-C, and CRP were located throughout the genome, mapping to 37, 30, 4, and 10 unique genes, respectively. When using the Illumina annotation files for gene mapping, we did not observe enrichment of GO terms or KEGG pathways for CpGs mediating the associations between stress and any cardiometabolic risk factor (FDR *q* < 0.01). However, when performing the gene-set analysis using eQTM data from FHS, we found that CpGs mediating the relationship between stress and each cardiometabolic risk factor were associated with the expression of genes enriched for several GO terms and KEGG pathways. CpGs that mediated the relationship between stress and BMI were associated with genes enriched for 10 GO terms and 2 KEGG pathways related to cytokine binding and receptor activity, cell surface, peptide receptor activity, and coreceptor activity (Additional file 2: Figure S5). Similarly, CpG mediators of the relationship between stress and WC were associated with genes enriched for 7 GO terms and 1 KEGG pathway related to cytokine binding and receptor activity, cell surface, and transmembrane signaling activity (Additional file 2: Figure S6). The mediating CpGs of the stress and HDL-C relationship were associated with genes enriched in 7 GO terms, including biological processes related to cytokine binding activity, cell surface, and neuron development (Additional file 2: Figure S7). We did not observe over-representation of KEGG pathways for these genes. Finally, the CpG mediators of the relationship between stress and CRP were associated with genes enriched for 11 GO terms and 2 KEGG pathways (Additional file 2: Figure S8), including pathways involving cytokine binding and receptor activity, cell surface, cell morphogenesis, and neuron development.

## Discussion

This study evaluated the role of epigenetic mediation on the relationship between cumulative psychosocial stress and cardiometabolic risk factors in a multi-racial/ethnic cohort of older adults. Epigenome-wide mediation analysis identified 50, 46, 7, and 12 CpG sites that mediated the total effect of stress on BMI, WC, HDL-C, and CRP, respectively. When reducing the dimensionality of the CpG mediators to their top uncorrelated PCs, the overall mediation effects across probes explained between 35.8% and 46.3% of these associations. Additionally, a number of independent CpGs were found to mediate the relationship between stress and each of the cardiometabolic risk factors, even after adjusting for all other mediating CpGs. Further adjustment of the mediation models for smoking slightly attenuated the mediation effects, indicating that smoking may be part of the pathway between stress and these risk factors; however, CpG mediators across all cardiometabolic risk factors remained nominally significant.

In epigenome-wide mediation analysis, we identified 32 distinct CpGs that mediated the association between psychosocial stress and multiple cardiometabolic risk factors. In particular, two CpGs, cg04803208 and cg25607249, mediated the relationships between stress and BMI, WC, HDL-C, and CRP, suggesting these methylation sites may play an important role on influencing cardiometabolic risk. Cg04803208 annotates to the *LOC100130298* gene on chromosome 8, which is a member of the long non-coding class of RNAs (lncRNA) [67]. LncRNAs play a critical role in gene expression through transcriptional, translational, and epigenetic regulation [68]. In large-scale EWAS, this CpG has been previously associated with type 2 diabetes, ischemic heart disease, and inflammatory Crohn’s disease [69, 70]. Cg25607249 maps to the gene body of the solute carrier family 1 member 5 (*SLC1A5*) gene, located on chromosome 19. *SLC1A5*, which encodes for the ASCT2 protein, is a sodium-dependent neutral amino acid transporter that plays a role in cellular glutamine homeostasis and is a critical regulator for cancer development and tumor growth [71, 72]. Prior research has established a link between *SLC1A5* and cardiovascular disease. Kennel et al. (2019) found that the mRNA levels and protein expression of *SLC1A5* in heart failure patients were suppressed compared to healthy controls [73]. Additionally, Westerman et al. (2019) discovered a region in *SLC1A5* (chr19:47287777–47288263) to be differentially methylated with respect to incident cardiovascular disease in the Women’s Health Initiative cohort, with results replicated in the Framingham Heart Study [74]. Interestingly, cg25607249, in addition to two other CpG mediators found in our HDMT results, cg24507742 (significant in WC and HDL models) and cg01406381 (significant in BMI and WC models), falls within this differentially methylated region, providing further evidence that these CpGs may be associated with cardiometabolic risk factors.

Several top CpGs reported in this study have been previously associated with CVD outcomes and their corresponding risk factors. Cg00420390, which was found to independently mediate the relationships between stress and BMI, WC, and CRP in the penalized-based mediation models, annotates to the mitotic arrest deficient 1 like 1 (*MAD1L1*) gene on chromosome 7. *MAD1L1* is a mitotic spindle assembly checkpoint gene that plays a critical role in cell cycle regulation by inhibiting the transition to anaphase until all chromosomes are properly aligned during metaphase [75]. This gene has been linked to a number of psychiatric conditions, including anxiety and depressive phenotypes [76, 77], schizophrenia [78], and bipolar disorder [79]. Additionally, emerging research has found *MAD1L1* to be a potential candidate gene for cardiovascular disease [80, 81].

Another CpG that independently mediated the relationships between stress and both BMI and WC, cg02370100, maps to the ATP binding cassette subfamily G member 1 (*ABCG1*) gene on chromosome 21. *ABCG1* is a transporter gene that plays a critical role in cellular lipid regulation by promoting the outflow of cholesterol to high-density lipoproteins [82]. Further, this gene participates in glucose metabolism by regulating insulin secretion. Due to its significant involvement in cholesterol homeostasis, *ABCG1* has been identified as a potential therapeutic target for atherosclerotic CVD [83]. Large-scale EWAS found cg02370100 to be associated with type 2 diabetes [69], serum triglycerides [84], and fasting insulin [85].

A subset of the mediating CpG eQTMs were associated with the expression of genes enriched in cytokine activity, including cytokine binding, cytokine receptor activity, and cytokine–cytokine receptor interaction. These findings support existing evidence that the upregulation of inflammatory cytokines predicts both cardiovascular disease and its cardiometabolic risk factors [86, 87]. Wu et al. (2020) found that obesity is associated with low-grade inflammation resulting from an increase in inflammatory cytokines in a variety of tissues, including adipose, skeletal muscle, and brain [88]. In turn, this inflammation has been thought to cause obesity-linked metabolic dysregulation, leading to insulin resistance and type 2 diabetes. Additionally, prior research has shown that chronic stress increases inflammatory cytokine activity in both animals and humans [89–91]; however, the association between stress and inflammatory pathways, particularly involving cytokines, is complicated, and more research needs to be done to better elucidate that relationship [92]. Overall, this study provides evidence that the methylation of inflammatory genes may mediate the relationship between psychosocial stress and cardiometabolic risk factors through mechanisms related to cytokine activity.

Additionally, CpG mediators of the relationships between stress and both HDL-C and CRP were associated with expression of genes enriched in neuron development, including cell morphogenesis involved in neuron differentiation, axon development, synapse organization, and axonogenesis. Chronic stress has been found to influence neuronal structure and function through the release of glucocorticoids (GCs), which are hormones secreted from the adrenal gland in response to stress [93]. Although GCs normally regulate themselves through negative feedback mechanisms to restore homeostasis, elevated GC levels due to prolonged stress exposure provoke structural remodeling of neurons and synaptic structure [94]. Further, hypersecretion of GCs due to chronic stress has been found to negatively impact health outcomes, including increased cardiovascular risk [95]. Future research should be conducted to better characterize the pathways of genes related to neuron development involved in mediating the relationship between stress and cardiometabolic risk factors.

Due to the novelty of our research question, we were unable to replicate our findings in an independent cohort of multi-ancestry older adults. However, we did observe overlap between our findings and those of Wang et al. (2022), which examined the epigenetic mediation of the relationship between neighborhood disadvantage, a single psychosocial stressor, and cardiovascular disease risk factors [29]. Specifically, two CpGs (cg02508743 and cg09315878) that were identified as mediators of the relationship between cumulative psychosocial stress and BMI in our study (*p* = 1.2E−06 and *p* = 2.7E−06, respectively) were also found to mediate the relationship between adult socioeconomic status and BMI in Wang et al. (*p* = 3.5E−09 and *p* = 3.9E−06, respectively). Both cg02508743, which maps to the *LYN* proto-oncogene on chromosome 8, and cg09315878, which maps to the stromal cell derived factor 4 (*SDF4*) gene on chromosome 1, have been discovered in multiple EWAS of BMI and maternal obesity [96–99]. As a result, these methylation sites should be considered in future studies to confirm their role in the epigenetic mediation of psychosocial stressors and cardiometabolic risk.

There are limitations to this study that should be acknowledged. First, the cumulative psychosocial stress score was comprised of only self-reported stress measures, most of which captured stress retrospectively (i.e., childhood adversity, lifetime trauma, stressful life events). Because of the time lag between when participants may have experienced the stressful event and when they were administered the survey, there is potential for differential misclassification of the stress exposure, where individuals who experienced greater cumulative stress across the life course may be more likely to report past stressors than those who were relatively unaffected by stress. Furthermore, some of the downstream health consequences of psychosocial stress, including depression and post-traumatic stress disorder, may influence the likelihood of a participant endorsing a stress exposure [100]. To get a more comprehensive definition of psychosocial stress, future analyses should include additional measures of stress, as well as objective biological markers such as cortisol, alpha-amylase, and pro-inflammatory cytokines [101]. Additionally, the cardiometabolic risk factors examined in this study are often on the same disease pathway [102–105], suggesting that the CpG mediators identified in epigenome-wide mediation analysis are likely not independent for each outcome.

Due to the short timeframe between the measurement of psychosocial stress, DNA methylation, and cardiometabolic risk factors, and because DNA methylation and cardiometabolic risk factors assessed through the VBS were measured concurrently (2016), we cannot confidently assert the causal direction of effect between the exposure, mediator, and outcome variables. As additional epigenetic, blood biomarker, and cardiometabolic risk factor data become available in HRS and other studies, future analyses should assess measures across multiple time points to establish a temporal relationship between psychosocial stress and cardiometabolic risk factors mediated by the epigenome.

Finally, CpG mediators that are in close proximity to one another and on the same causal pathway may be highly correlated. However, most mediation methods that examine high-dimensional DNA methylation data, including HDMT, do not explicitly model the correlation among potential CpG mediators and instead assess their contributions univariately [106]. Because we were interested in the epigenetic mediation of the association between stress and cardiometabolic risk factors across the epigenome, we did not have the computational power to account for the correlations between the nearly 800,000 CpGs assessed in high-dimensional mediation analyses. To address this limitation, we conducted PC analysis to characterize the overall mediation effect across identified CpGs in HDMT and account for possible correlation among those CpG sites [29].

This study had several key strengths. To our knowledge, this was the first study to utilize a high-dimensional mediation testing method, HDMT, to formally test the mediation effect of CpGs across the epigenome on the relationship between cumulative stress and cardiometabolic risk factors. This method used a corrected mixture reference distribution utilizing the composite structure of the null hypothesis, thus providing well-calibrated p values under the null. Further, we utilized a composite psychosocial stress score that captured several stress domains across the life course, allowing us to assess the cumulative impact of stress on DNA methylation and subsequent cardiometabolic risk factors, as opposed to the influence of a single stressor. Lastly, this research was conducted in a multi-racial/ethnic population of European, African, and Hispanic/Latino participants, which allowed us to examine the epigenetic mediation of psychosocial stress on cardiometabolic risk in a diverse population.

## Conclusion

In summary, results from this study demonstrate that DNA methylation partially mediates the relationship between cumulative psychosocial stress and several cardiometabolic risk factors. Furthermore, a subset of the mediating CpG eQTMs found in the epigenome-wide analysis were associated with expression of genes enriched in pathways related to cytokine binding and receptor activity, as well as neuron development. Future studies should be conducted to replicate these findings and further elucidate the mechanisms through which DNA methylation mediates the effect of psychosocial stress on cardiometabolic risk.

## Supplementary Information

Additional file1: This file (.xlsx) contains supplementary table information (Tables S1-S16).

Additional file2: This file (.docx) contains supplementary table information (Figures S1-S8).

## Data Availability

Health and Retirement Study (HRS) survey data are publicly available at https://hrs.isr.umich.edu/data-products and can be accessed upon request by registered users who meet security requirements and agree to the specified data use conditions. HRS DNA methylation data are available at the National Institute on Aging Genetics of Alzheimer’s Disease Data Storage Site (NIAGADS, accession number: NG00153).

## Abbreviations

BMI: Body mass index
CGI: CpG islands
CRP: C-reactive protein
CVD: Cardiovascular disease
DBP: Diastolic blood pressure
DHS: DNase I hypersensitivity sites
eQTM: Expression quantitative trait methylation
EWAS: Epigenome-wide association study
FDR: False discovery rate
FHS: Framingham Heart Study
GC: Glucocorticoid
GO: Gene ontology
HDL-C: High-density lipoprotein cholesterol
HDMT: High-dimensional mediation testing
HRS: Health and Retirement Study
IQR: Interquartile range
KEGG: Kyoto Encyclopedia of Genes and Genomes
LDL-C: Low-density lipoprotein cholesterol
lncRNA: Long non-coding class of RNAs
PC: Principal component
SBP: Systolic blood pressure
TC: Total cholesterol
TG: Triglycerides
VBS: Venous blood study
WC: Waist circumference
