# Supplementary material for: Epigenome-wide mediation analysis of the relationship between psychosocial stress and cardiometabolic risk factors in the Health and Retirement Study (HRS)
Source: Clin Epigenetics. 2024 Dec 18;16:180. doi: 10.1186/s13148-024-01799-4 (PMC11656575; doi:10.1186/s13148-024-01799-4)
Supplement: Supplementary file 2 — Additional file2: This file (.docx) contains supplementary table information (Figures S1-S8). [file 13148_2024_1799_MOESM2_ESM.docx]

**Figure S1.** (A) Manhattan plot of the epigenetic mediation effects from the association between psychosocial stress and BMI using the high-dimensional mediation testing method. The green dots represent CpGs that meet the epigenome-wide significance threshold (FDR q<0.05). (B) Quantile-quantile plot of the epigenetic mediation effects from the association between psychosocial stress and BMI (inflation factor λ=1.03)

1. **
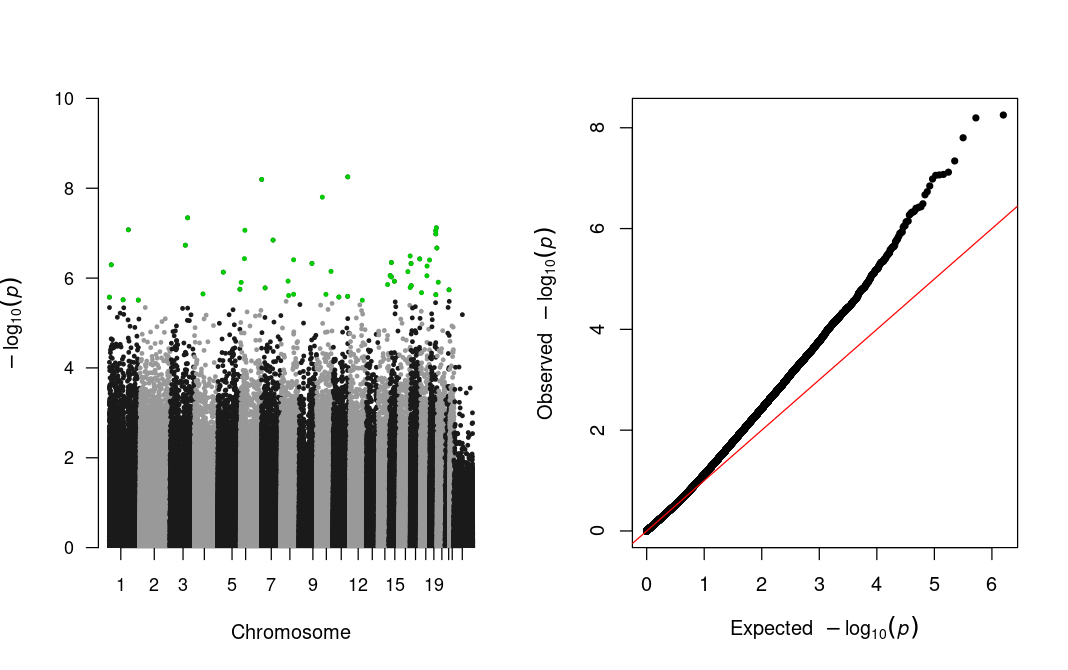
 (B)**

**44**

**Figure S2.** (A) Manhattan plot of the epigenetic mediation effects from the association between psychosocial stress and WC using the high-dimensional mediation testing method. The green dots represent CpGs that meet the epigenome-wide significance threshold (FDR q<0.05). (B) Quantile-quantile plot of the epigenetic mediation effects from the association between psychosocial stress and WC (inflation factor λ=1.25)

1. **(B)**

**
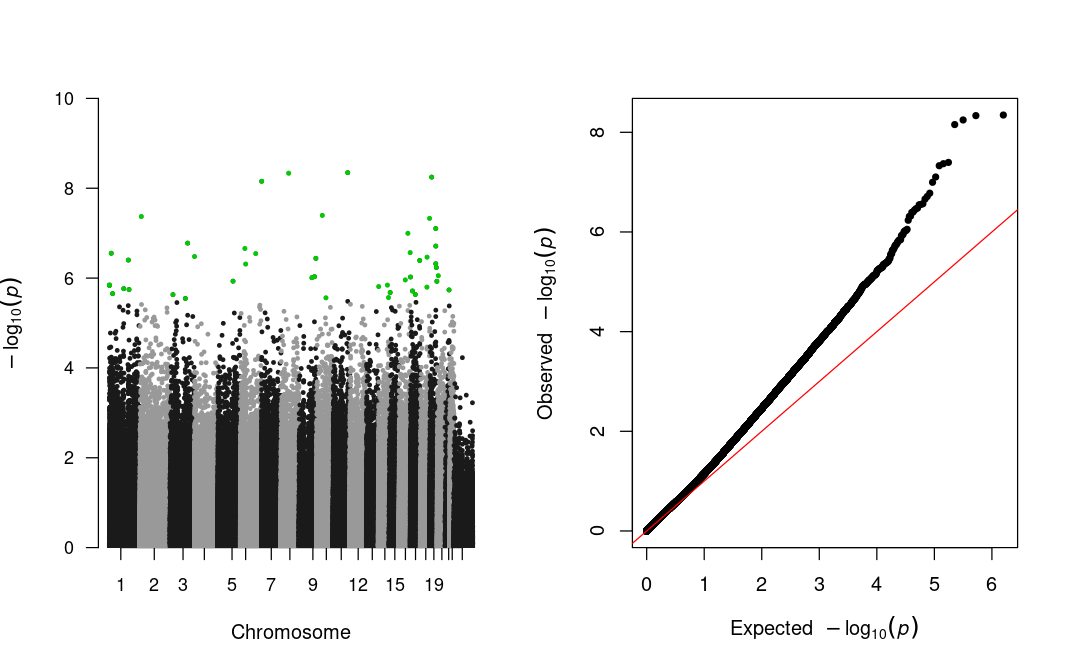
**

**Figure S3.** (A) Manhattan plot of the epigenetic mediation effects from the association between psychosocial stress and HDL-C using the high-dimensional mediation testing method. The green dots represent CpGs that meet the epigenome-wide significance threshold (FDR q<0.05). (B) Quantile-quantile plot of the epigenetic mediation effects from the association between psychosocial stress and HDL-C (inflation factor λ=0.92)

1. **(B)**

**
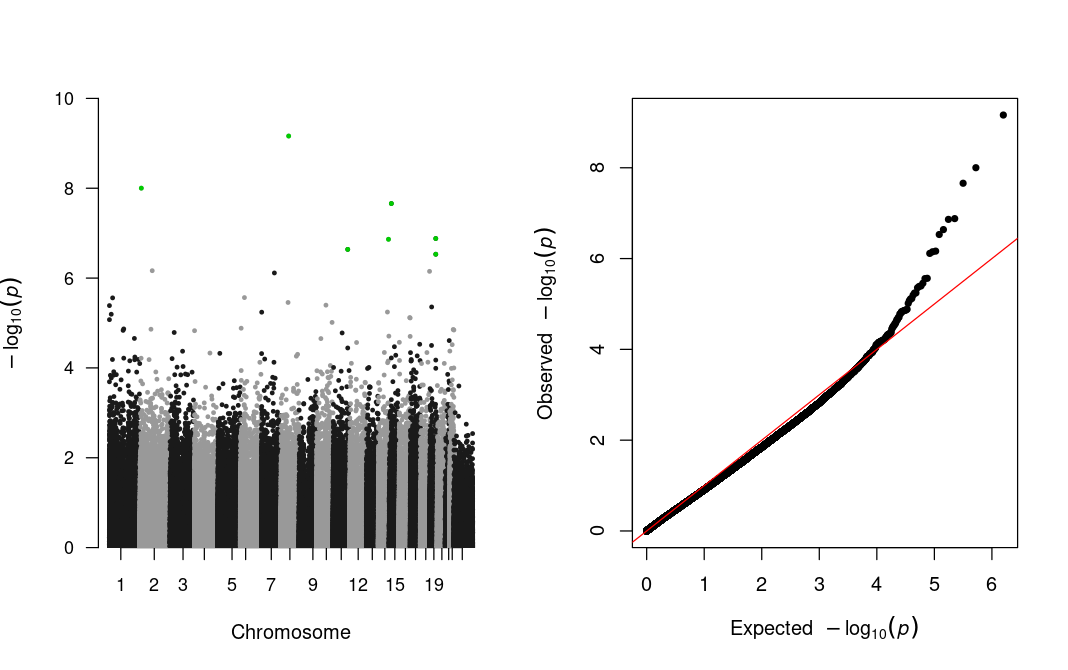
**

**Figure S4.** (A) Manhattan plot of the epigenetic mediation effects from the association between psychosocial stress and CRP using the high-dimensional mediation testing method. The green dots represent CpGs that meet the epigenome-wide significance threshold (FDR q<0.05). (B) Quantile-quantile plot of the epigenetic mediation effects from the association between psychosocial stress and CRP (inflation factor λ=0.93)

1. **(B)**

**
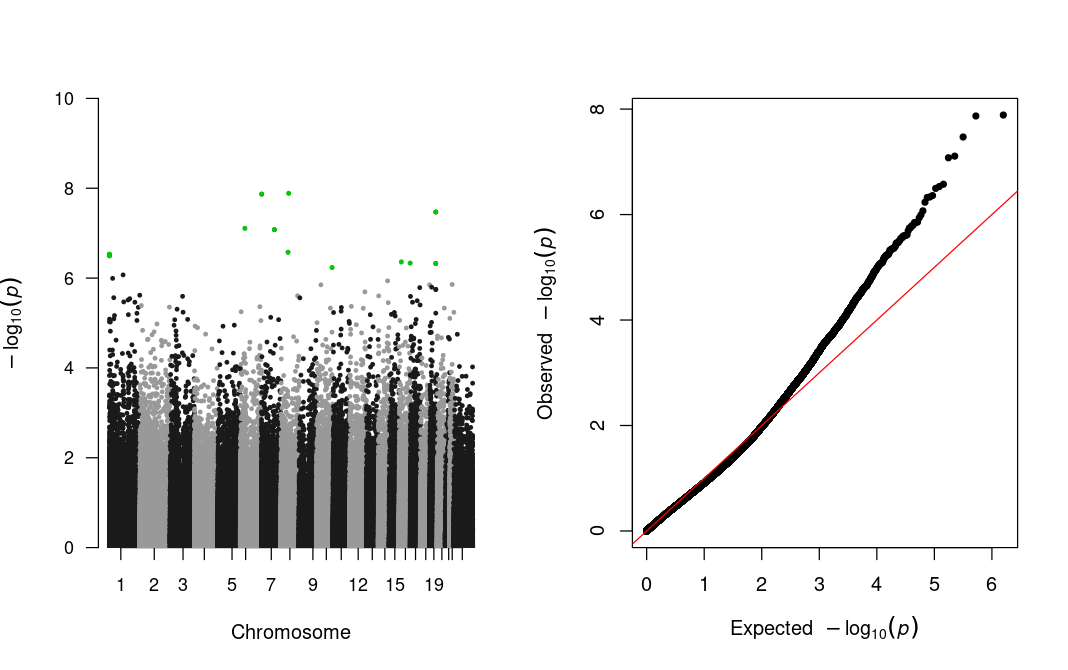
**

**Figure S5**. (A) Dotplot showing the Gene Ontology (GO) biological processes and molecular function enrichment results for genes whose expression was associated with CpGs mediating the relationship between stress and BMI identified in high-dimensional mediation testing (Model 1) (B) Dotplot showing the KEGG pathway enrichment results for genes whose expression was associated with CpGs mediating the relationship between stress and BMI identified in high-dimensional mediation testing (Model 1).


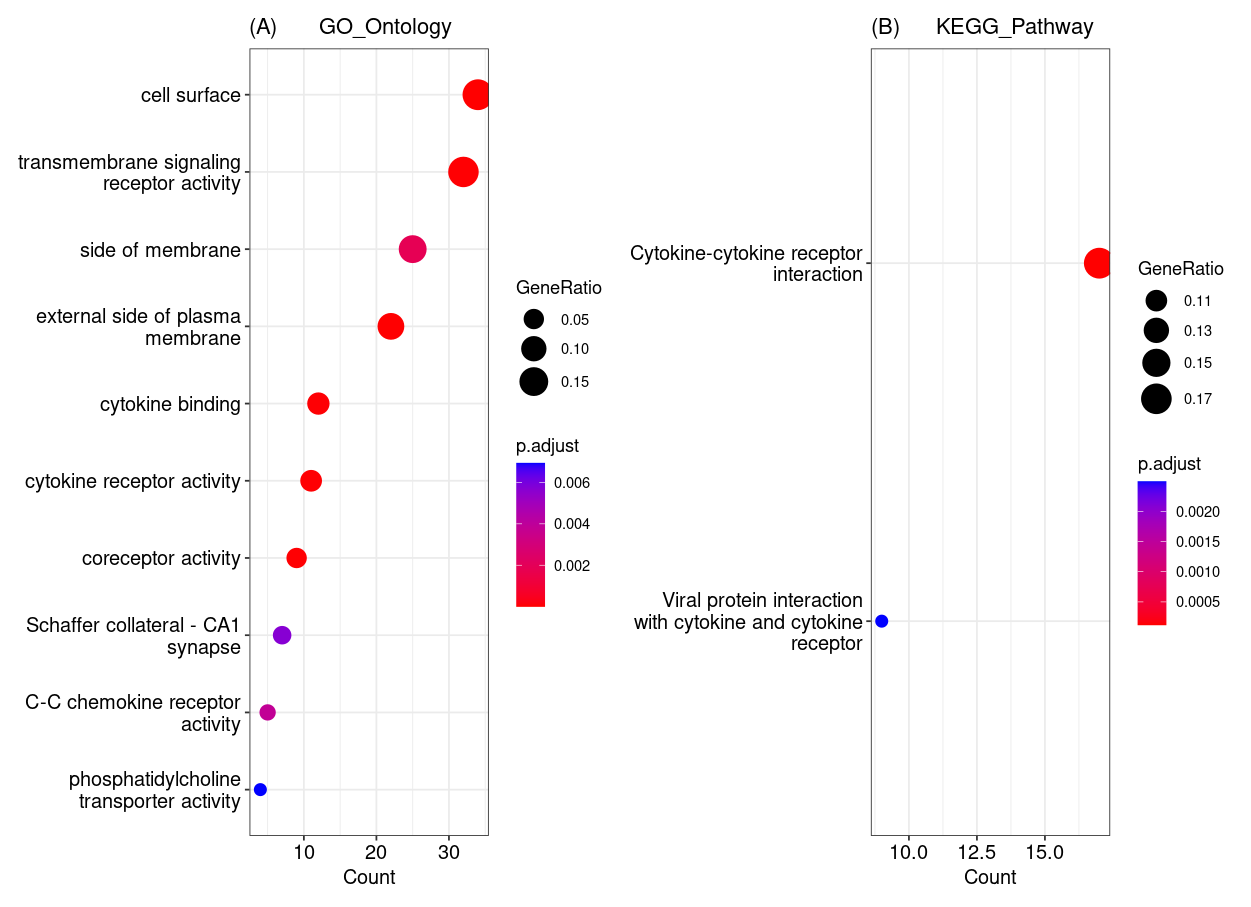


Dot size represents the fraction of differentially expressed genes found in the gene set. Dot color represents the FDR q-value, with a significance level of FDR q<0.01.

**Figure S6**. (A) Dotplot showing the Gene Ontology (GO) biological processes and molecular function enrichment results for genes whose expression was associated with CpGs mediating the relationship between stress and WC identified in high-dimensional mediation testing (Model 1) (B) Dotplot showing the KEGG pathway enrichment results for genes whose expression was associated with CpGs mediating the relationship between stress and WC identified in high-dimensional mediation testing (Model 1).

**
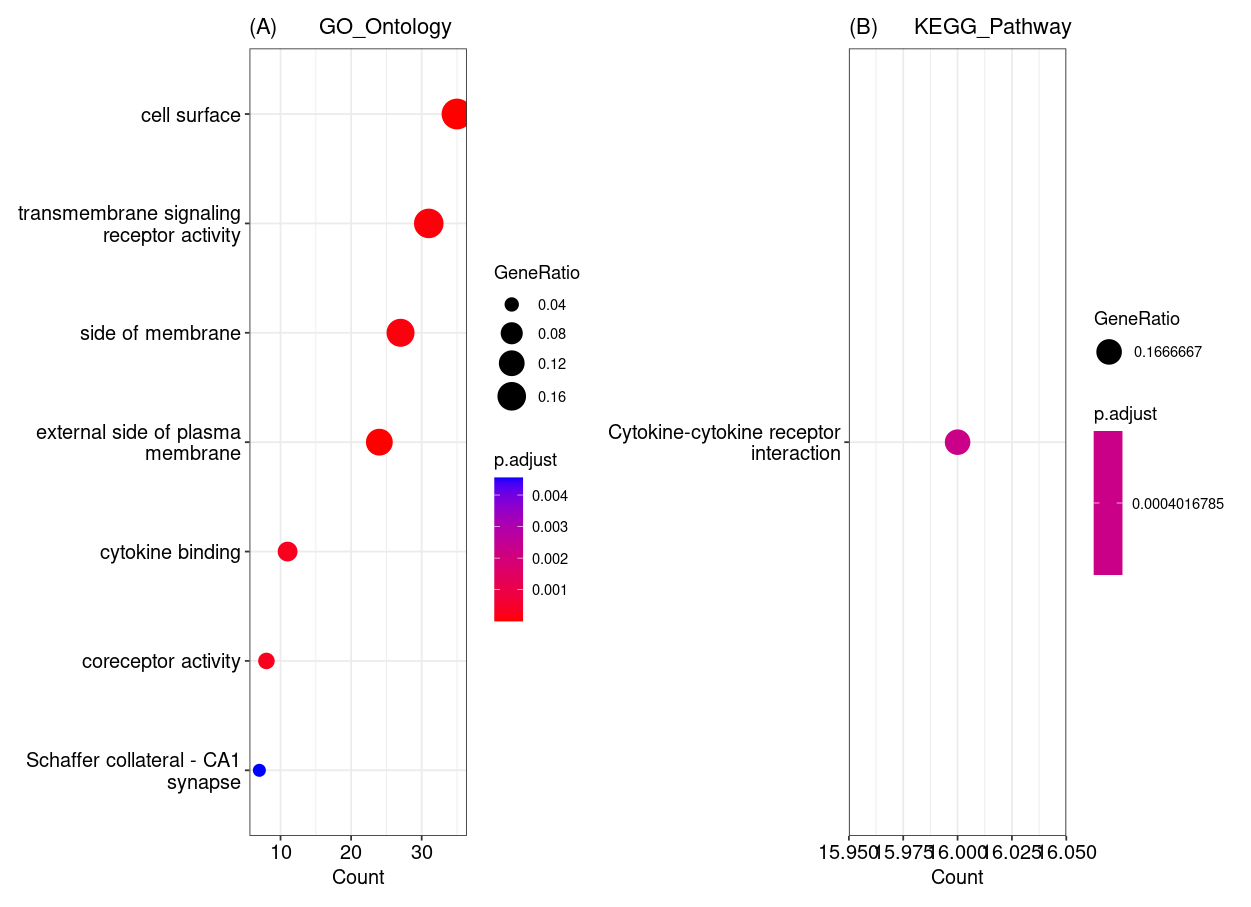
**

Dot size represents the fraction of differentially expressed genes found in the gene set. Dot color represents the FDR q-value, with a significance level of FDR q<0.01.

**Figure S7**. Dotplot showing the Gene Ontology (GO) biological processes and molecular function enrichment results for genes whose expression was associated with CpGs mediating the relationship between stress and HDL-C identified in high-dimensional mediation testing (Model 1).


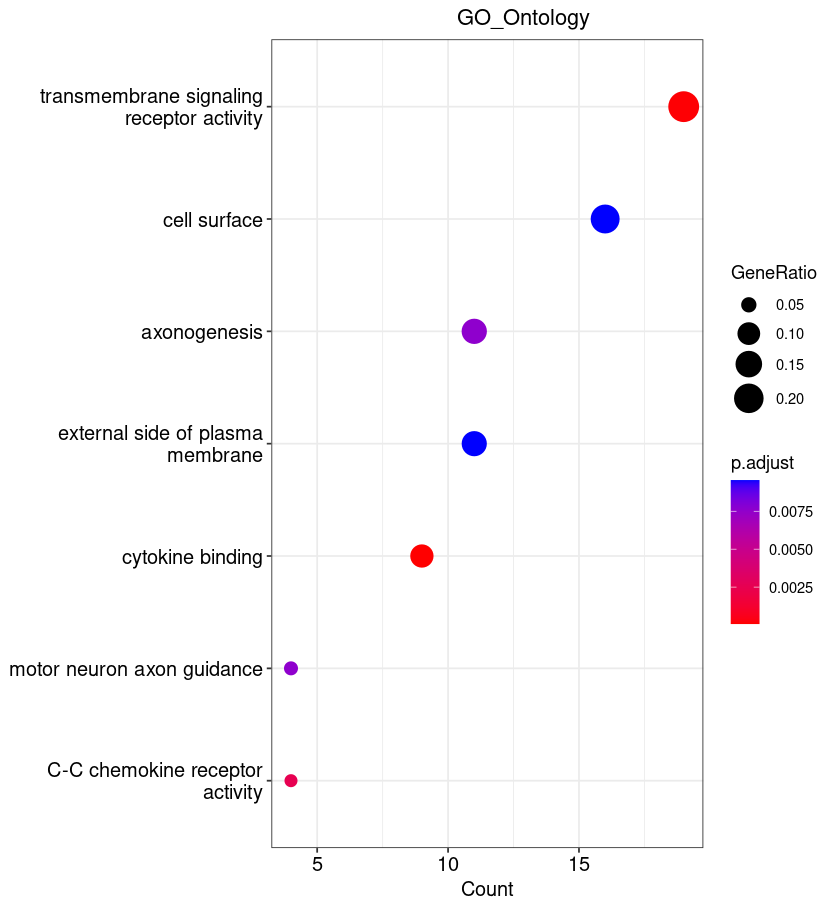


Dot size represents the fraction of differentially expressed genes found in the gene set. Dot color represents the FDR q-value, with a significance level of FDR q<0.01.

**Figure S8**. (A) Dotplot showing the Gene Ontology (GO) biological processes and molecular function enrichment results for genes whose expression was associated with CpGs mediating the relationship between stress and CRP identified in high-dimensional mediation testing (Model 1) (B) Dotplot showing the KEGG pathway enrichment results for genes whose expression was associated with CpGs mediating the relationship between stress and CRP identified in high-dimensional mediation testing (Model 1).


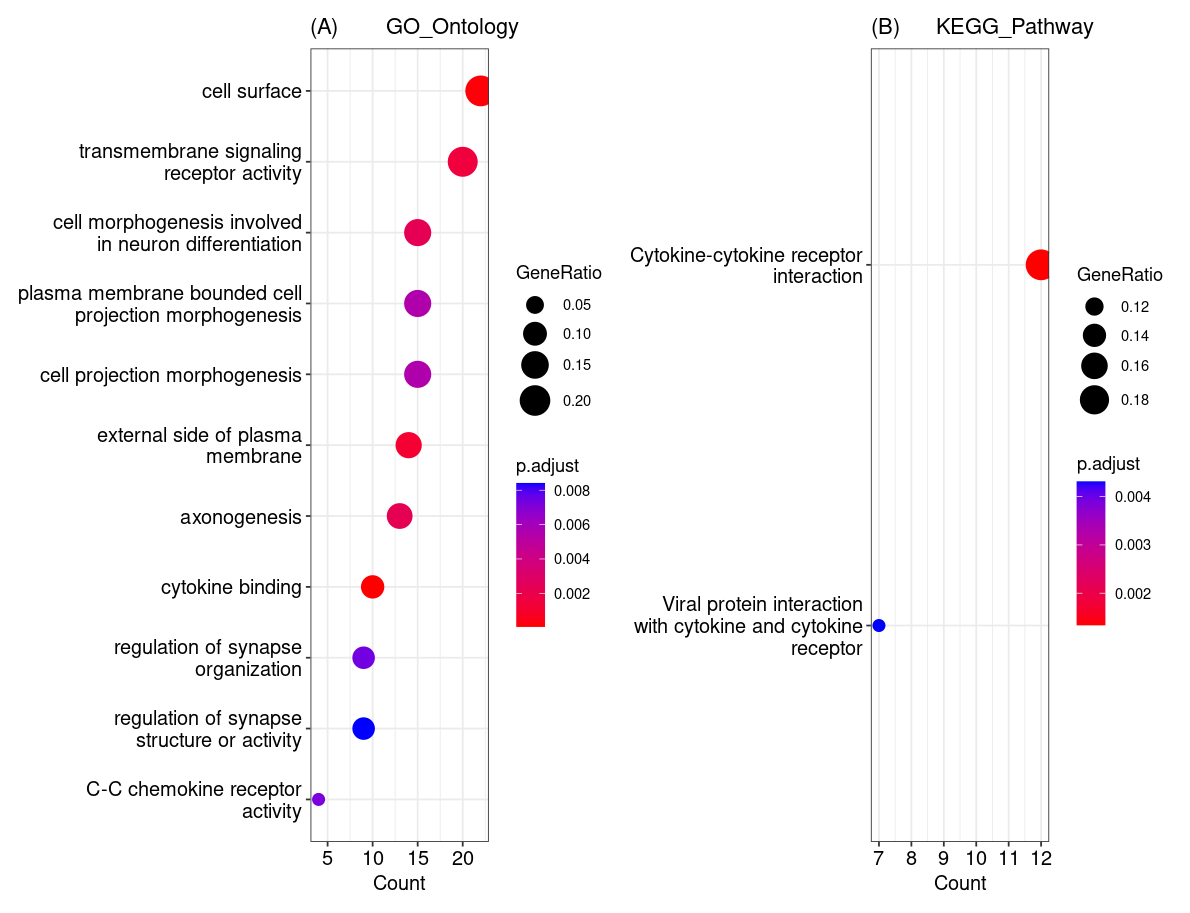


Dot size represents the fraction of differentially expressed genes found in the gene set. Dot color represents the FDR q-value, with a significance level of FDR q<0.01.
